# The level of depressive, anxiety and obsessive-compulsive psychopathological dimensions in symptomatic versus asymptomatic SARS-CoV-2 infected pregnant women

**DOI:** 10.1192/j.eurpsy.2022.1372

**Published:** 2022-09-01

**Authors:** V.R. Enatescu, M. Dinescu, R. Kalinovic, G. Vlad

**Affiliations:** 1 University of Medicine and Pharmacy “Victor Babes” Timisoara, Psychiatry, Timisoara, Romania; 2 “Pius Brinzeu” Emergency Clinical County Hospital Timisoara, “eduard Pamfil” Clinic Of Psychiatry, Timisoara, Romania; 3 “Victor Babes” University of Medicine and Pharmacy, Biochemistry, TIMISOARA, Romania

**Keywords:** depression, anxiety, obsessive-compulsive, SARS-CoV-2

## Abstract

**Introduction:**

The neurotropic valence of SARS-CoV-2 has been revealed in several studies. Depressive and anxiety symptoms are more frequent in the perinatal period leading to maternal and neonatal negative outcomes. Accordingly, depressive and anxiety symptoms are more frequent in the perinatal period leading to negative consequences in both mothers and their neonates.

**Objectives:**

To determine the level of depression, anxiety, and obsessive-compulsive symptoms depending on the severity of SARS-CoV-2 infection of pregnant women.

**Methods:**

Based on the RT-PCR test, thirty-eight pregnant women with SARS-CoV-2 infection, hospitalized in Bega Clinic in Timisoara, were assessed concerning the presence of psychopathology. The severity of infection was dichotomized based on the presence or absence of the symptoms. The Edinburgh Postnatal Depression Scale, State and Trait Anxiety Inventory, and the Obsessive-Compulsive Inventory were administered to all participants.

**Results:**

Of 38 recruited pregnant women, 12 (31,5%) had symptomatic SARS-CoV-2 infection. Symptomatic SARS-CoV-2 infected pregnant women had a higher average score of depression (p = 0.001) and state and trait anxiety (p = 0.002 and p < 0.001, respectively) compared to their asymptomatic counterparts. There were no differences in obsessive-compulsive symptoms (p > 0.05) in relation to the severity of SARS-CoV-2 infection.

**Conclusions:**

The SARS-CoV-2 infection significantly interferes with the psychological status, thus jeopardizing the mental health of pregnant women. Therefore, SARS-CoV-2 infection should be considered an additional risk factor for anxiety and affective disorders during pregnancy.

**Disclosure:**

No significant relationships.

